# Antioxidant Compounds from Edible Mushrooms as Potential Candidates for Treating Age-Related Neurodegenerative Diseases

**DOI:** 10.3390/nu15081913

**Published:** 2023-04-15

**Authors:** Grazia Maria Liuzzi, Tania Petraglia, Tiziana Latronico, Aniello Crescenzi, Rocco Rossano

**Affiliations:** 1 Department of Biosciences, Biotechnologies and Environment, University of Bari “Aldo Moro”, 70126 Bari, Italy; tiziana.latronico@uniba.it; 2 Department of Sciences, University of Basilicata, 85100 Potenza, Italy; taniapetraglia.5@gmail.com; 3 School of Agricultural, Forestry, Food and Environmental Sciences, University of Basilicata, 85100 Potenza, Italy; aniello.crescenzi@unibas.it

**Keywords:** edible mushrooms, antioxidant, healthy aging, age-related neurodegenerative diseases, Alzheimer’s disease, Parkinson’s diseases

## Abstract

The last century has seen an increase in our life expectancy. As a result, various age-related diseases, such as neurodegenerative diseases (NDs), have emerged, representing new challenges to society. Oxidative stress (OS), a condition of redox imbalance resulting from excessive production of reactive oxygen species, represents a common feature that characterizes the brains of elderly people, thus contributing to NDs. Consequently, antioxidant supplementation or dietary intake of antioxidant-containing foods could represent an effective preventive and therapeutic intervention to maintain the integrity and survival of neurons and to counteract the neurodegenerative pathologies associated with aging. Food contains numerous bioactive molecules with beneficial actions for human health. To this purpose, a wide range of edible mushrooms have been reported to produce different antioxidant compounds such as phenolics, flavonoids, polysaccharides, vitamins, carotenoids, ergothioneine, and others, which might be used for dietary supplementation to enhance antioxidant defenses and, consequently, the prevention of age-related neurological diseases. In this review, we summarized the role of oxidative stress in age-related NDs, focusing on the current knowledge of the antioxidant compounds present in edible mushrooms, and highlighting their potential to preserve healthy aging by counteracting age-associated NDs.

## 1. Introduction

Aging is a physiological process characterized by a progressive decline in the physical functions of the body, influenced by genetic, epigenetic, environmental, and social factors. Several aging mechanisms identified so far include the accumulation of cellular senescence, genomic instability, impaired protein homeostasis, shortening of telomeres, mitochondrial dysfunction, dysregulated nutrient sensing, and altered cellular communication [1]. As a result of the increasing life expectancy in the world, various age-related diseases have emerged, representing a heavy economic and psychological burden not only for patients but also for their families and for society [2]. Among age-related diseases, neurodegenerative disorders (ND) are noteworthy due to their devastating nature and lack of effective therapies. The most common age-related ND are Alzheimer’s disease (AD) and Parkinson’s disease (PD). These neurodegenerative diseases are characterized by a progressive and gradual loss of neural tissue or neurons with a consequent impairment of motor or cognitive functions, and by the deposition of abnormally aggregated proteins in brain tissue [3,4].

Although each of these pathologies affect different areas of the central nervous system (CNS) and exhibit different clinical manifestations, both AD and PD share common etiopathogenetic pathways. A common feature of neurodegenerative diseases is oxidative stress (OS), a condition of redox imbalance resulting from excessive production of reactive oxygen species (ROS) [5]. A vast amount of experimental evidence indicates that bioenergetic impairments and alterations in oxidation-reduction homeostasis are present in the brains of elderly people [6]. The brain is particularly susceptible to oxidative damage due to the high oxygen consumption and the high glucose turnover rate as well as high levels of redox-active iron in some regions. Furthermore, the brain contains low levels of antioxidant enzymes such as glutathione (GSH) peroxidase (GPx), catalase (CAT), and superoxide dismutase (SOD), and also of non-enzymatic endogenous antioxidants such as GSH and vitamin E [7]. As age progresses, the endogenous antioxidant systems become less efficient, and this is one of the reasons why elderly people are more susceptible to oxidative stress. Therefore, antioxidant supplementation represents an effective preventive and therapeutic intervention to maintain the integrity and survival of neurons and to counteract the pathologies associated with aging.

Recent studies indicate that dietary interventions have the potential to prevent and even improve age-related neurological decline [8]. Decades of research through in vitro and animal model studies as well as clinical trials have highlighted the ability and efficacy of natural antioxidants to improve behavioral outcomes by inducing physiological and biochemical changes that protect the brain by reducing risk factors associated with the development of NDs. For thousands of years, mushrooms have been used for their nutritional value and medicinal properties [9]. They therefore represent not only a food but also a precious source of biologically active compounds that act as nutraceuticals. Numerous studies have shown that edible mushrooms possess anticancer, anti-atherosclerotic, hypocholesterolemic, hypolipidemic, antiviral, antimicrobial, immunostimulant, anti-inflammatory, antioxidant, and anti-aging effects [9,10,11,12]. The antioxidant properties of edible mushrooms are mainly related to their content in phenolic compounds and polysaccharides [10,11]. Among polyphenol groups, phenolic acids are the main antioxidants, whereas the major antioxidant effects of polysaccharides are attributed to -glycans. These compounds show significant ROS scavenging activity and are also able to stimulate the activity of SOD, CAT, and GPx enzymes [11]. In this review, we discussed the role of oxidative stress in age-related ND, highlighting the properties of antioxidant compounds present in edible mushrooms and their potential to preserve healthy aging.

## 2. Metodology

For this review, an extensive online search was conducted on edible mushrooms as a source of natural antioxidants in healthy aging, mainly in neurodegenerative diseases. Various important databases, such as Pubmed, Scopus, and ScienceDirect, were used for the literature survey, using the following keywords: edible mushrooms, antioxidant, polyphenols, phenolics, flavonoids, polysaccharides, vitamins, ergothioneine, and minerals in combination with healthy aging, neuroprotection, Alzheimer’s disease, and Parkinson’s disease.

## 3. Oxidative Stress

Oxygen is essential for aerobic organisms, but its metabolism inevitably leads to the formation of ROS. Oxygen is the final acceptor in the mitochondrial electron transport system (ETS), leading to the formation of ATP in the mitochondria. During the flow of electrons along the mitochondrial electron transport chain, some electrons can react directly with oxygen, generating ROS that, in turn, can react with various biological macromolecules, “stealing” electrons to restore their electroneutrality [12]. In eukaryotic cells, over 90% of ROS are generated by mitochondria. A second source of free radicals is represented by different oxidases such as xanthine oxidase (XO) and NADPH oxidases (NOX), multi-subunit enzyme complexes that are located in the cell membrane and expressed in CNS cells, including neurons and glial cells [13]. ROS is a general term that includes all reactive forms of O_2_, both the radical species such as the superoxide radical (O_2_^•−^) and the hydroxyl radical (OH^•^) as well as the non-radical species such as the hydrogen peroxide (H_2_O_2_) and the singlet oxygen (^1^O_2_), which can be converted into free radicals. ROS are small and highly unstable molecules, which play dual and opposite roles depending on the context. Indeed, when produced in moderate amounts, ROS are involved in physiological events such as proliferation, primary immune defense, cell differentiation, and signaling [14]. However, when both radical and non-radical ROS are produced in excess, they exert pro-oxidant activity against various biomolecules, triggering a sequence of chain reactions resulting in molecular and cellular damage [15]. Nitric oxide (NO) and radical species related to it as well as reactive nitrogen species (RNS) such as the highly reactive peroxynitrite (ONOO^−^) can also contribute to cell damage. At the cellular level, overproduction of ROS and RNS can result in harmful effects, including oxidation of DNA, RNA, and proteins, plus peroxidation of the polyunsaturated fatty acids of cell membranes. The oxidation of DNA and RNA may result in gene mutation and interruption of transcription. As a consequence of lipid peroxidation, cellular membranes may be damaged, thus compromising cell integrity and signaling. Protein oxidation may lead to the generation of misfolded and aggregated proteins. The balance between beneficial and detrimental effects of radical species is preserved by the activity of enzymatic and non-enzymatic molecules, which are known as endogenous detoxicant antioxidant systems [16].

## 4. Role of Oxidative Stress in Aging and Age-Related Neurodegenerative Diseases

**Figure 1:**
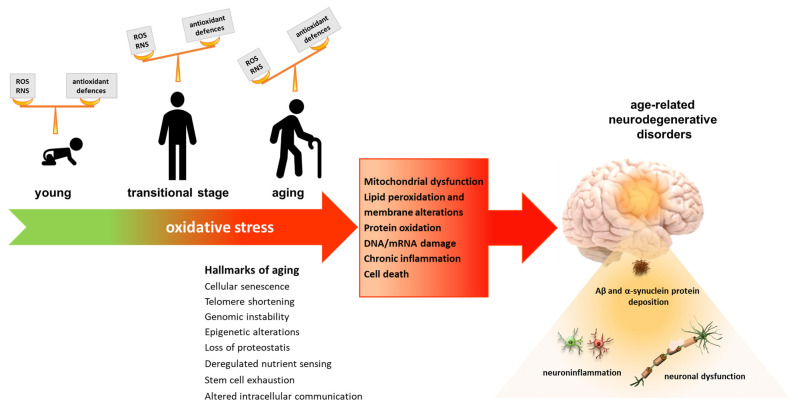
Role of oxidative stress in health, aging, and neurodegenerative diseases. In healthy conditions, levels of ROS/RNS are balanced by efficient mechanisms of defense. However, during aging, the oxidant levels increase, while the antioxidant defences become less efficient, generating an imbalance that leads to oxidative stress. This condition leads to oxidative damage of the main biomolecules, resulting in the development of age-related neurodegenerative conditions such as Alzheimer’s and Parkinson’s diseases.

Oxidative stress has been identified as a major risk factor associated with aging and the initiation and progression of age-related neurodegenerative diseases [5] (Figure 1). The high susceptibility of the brain towards oxidative stress and the consequent development of neurodegenerative processes depends on the fact that the CNS is a metabolically active organ, which requires about 20% of the total energy consumption of the organism. Therefore, to maintain its efficiency, brain tissue is rich in active mitochondria that produce high amounts of ROS [17]. Furthermore, the CNS presents high levels of polyunsaturated fatty acids and high levels of transition metals, required by many mitochondrial enzymes, which catalyze the synthesis of free radicals. Another important source of free radicals in the CNS is also represented by the metabolism of neurotransmitters. In addition to this, a further element that contributes to making the CNS extremely susceptible to oxidative stress is represented by the low level of antioxidant enzymes such as SOD, CAT and GPx, and non-enzymatic antioxidants such as vitamin E and GSH [7].

Currently, AD and PD diseases, affecting millions of people worldwide, represent the most prevalent age-related neurodegenerative disorders, constituting a major health problem for aging societies.

### 4.1. Alzheimer’s Disease

Alzheimer’s disease is the main cause of dementia. It is characterized by a severe atrophy of the cerebral cortex caused by neuron death and synaptic degeneration, and it is manifested by psychological, motor, and memory impairment [18]. Hallmarks of AD are extracellular amyloid plaques formed by an aggregation of amyloid-beta peptide (Aβ) and the presence of intracellular tau neurofibrillary tangles (NFT) and neuroinflammation [19,20]. Oxidative stress represents a key player in the pathogenesis of AD [21], since it contributes to Aβ production and plaque apposition, but, at the same time, extracellular Aβ accumulation and impaired mitochondrial function could trigger free radical formation [22]. Increased ROS levels also contribute to problems ranging from the hyperphosphorylation of Tau to the formation of neurofibrillary tangles to cell death [5]. Another mechanism by which oxidative stress influences AD could be the imbalance of bioactive metals [23]. In fact, the post-mortem analysis of brains of AD patients showed an accumulation of iron, zinc, and copper in Aβ plaques, and the ability of these metals to promote the aggregation of Aβ [24] and the hyperphosphorylation of Tau [25]. Finally, amyloid, Tau protein, and ROS affect the activity and uptake of the glutamate receptor by exacerbating Ca^2+^ influx into postsynaptic neurons, thus leading to an oxidative condition that ultimately determines the synaptic dysfunction responsible for AD [26,27]. In several studies, an increase in oxidative and nitrosative stress markers has been demonstrated in post-mortem samples from different areas of the cerebral cortex of AD and in patients affected by mild cognitive impairment (MCI) [28,29], reinforcing the idea that ROS and RNS play a key role in disease progression. Lipid peroxidation also seems to play a key role in the pathogenesis of AD [30]. In fact, increased levels of various lipid peroxidation products, such as 4-hydroxynonenal (HNE), malondialdehyde (MDA), and F(2)/F(4)-isoprostane, have been detected in the plasma, CSF, and brains of subjects with AD and MCI [31,32,33], which also suggests the possibility of using lipid peroxidation products as markers for AD identification. Finally, increased levels of advanced glycation end-products (AGEs) and their receptors have been evidenced in the cerebrospinal fluid and the microglia cells of the brains of AD patients [34], suggesting their contribution to neurofibrillary tangles and senile plaque formation. Moreover, numerous studies have evidenced decreased levels of endogenous antioxidant enzymes in the brain of AD patients from the early stages of the disease [35], suggesting that the alteration of the redox balance is due not only to an increase in the production of ROS/RNS but also to a lower efficiency of the endogenous antioxidant systems.

### 4.2. Parkinson’s Disease

Parkinson’s disease is the second most common neurodegenerative disorder resulting from progressive degeneration of the dopaminergic neurons in the substantia nigra (SN) pars compacta associated with microglia activation. The main pathological hallmarks of PD are intracellular inclusions that contain aggregates of α-synuclein (α-Syn) (Lewy bodies). The motor symptoms of PD in patients include weakness, tremor, rigidity, bradykinesia, and postural instability [36,37]. In PD, the oxidative stress is associated with the aggregation of the presynaptic protein α-Syn and the formation of Lewy bodies. Elevated levels of oxidative and nitrosative stress markers such as 4-HNE, carbonyl protein, 8-hydroxy-2′-deoxyguanosine (OH8dG), and 8-hydroxyguanosine were detected in serum, cerebrospinal fluid, and postmortem specimens of the brains of patients suffering from PD. [38,39,40,41]. Similarly, α-sin nitration levels were increased in the brain of PD patients [42]. The role of oxidative stress in the sequence of events leading to the degeneration of dopaminergic neurons is also due to the apoptosis of dopaminergic brain cells triggered by activation of the p38 mitogen-activated protein kinase pathway [43]. In addition, elevated levels of lipid peroxidation markers have been detected in the post-mortem brains of PD patients [44]. High levels of MDA linked to α-synuclein have been evidenced in the frontal cortex of PD cases, supporting the hypothesis that lipid peroxidation may precede and contribute to α-Syn aggregation [45]. Glycated protein levels were also significantly higher in the cerebral cortex of PD patients than in the controls [46]. Similarly, the increase in the concentration of AGE was found to be related to the death of dopaminergic neurons. Antioxidant status was also found to be significantly changed in PD compared with age-matched healthy subjects. In particular, SOD levels were increased in the basal ganglia and CNS of patients with PD [47], while decreased GSH levels were observed [48]. Finally, an accumulation of iron in the substantia nigra of PD patients has also been found to correlate with the spatiotemporal progression of neuronal loss [5] and increase the oxidation or phosphorylation of α-Syn in the substantia nigra [49].

## 5. Mushrooms as Sources of Antioxidant Compounds

**Figure 2:**
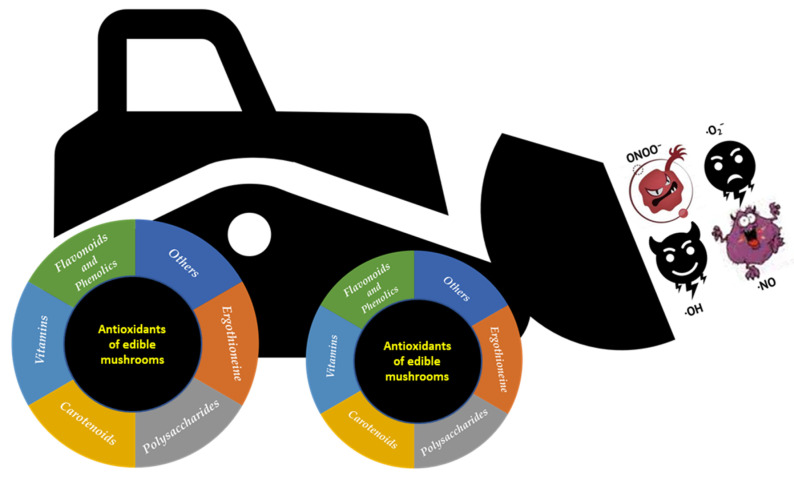
The antioxidant mycochemicals of edible and medicinal mushrooms.

Mushrooms belonging to the phylum *Basidiomycota* include around 2000 edible/medicinal species, but only a few dozen of them are currently cultivated commercially in the world [50]. Among the cultivated species, the most commercially important are *Agaricus bisporus* (button), *Lentinula edodes* (shiitake), *Boletus edulis* (porcino), *Pleurotus eryngii* (king oyster mushroom), *Pleurotus ostreatus* (oyster mushroom), *Agrocybe aegerita* (pioppino), *Flammulina velutipes* (enoki), *Grifola frondosa* (maitake), *Volvariella volvacea* (paddy straw mushroom), *Calocybe indica* (milky mushroom), and *Hericium erinaceus* (lion’s mane mushroom) [51]. The top five of the most cultivated mushrooms worldwide is composed by five genera: *Agaricus*, *Lentinus*, *Pleurotus*, *Flammulina*, and *Auricularia*, accounting for 85% of the cultivated edible mushrooms. In the last decade, especially, humans consumed several mushroom species for health benefits, and thus improved the commercial cultivation demand and the global markets [52,53]. Edible mushrooms, including those wild-harvested or cultivated fungal species, possess excellent organoleptic properties and high nutritional values. Their nutritional composition shows a wide variability among the different species [54]. Mushrooms are a low calorie food (~28–35 kcal/100 g), rich in fibers and poor in fats, and, in addition, they are gluten- and cholesterol-free. The fruiting bodies contain around 85–90% water, whereas their dry matter have approximately 50–65% of total carbohydrates, 19–35% proteins, and 2–6% fat. Mushrooms are a good source of unsaturated fatty acids, vitamins (B1, B2, B12, C, D), and high-quality proteins as they contain all the essential amino acids. The minerals found in mushrooms are principally potassium, phosphorus, calcium, iron, manganese, magnesium, zinc, and selenium [55]. In addition to their culinary properties, mushrooms are a precious source of biologically active compounds that act as nutraceuticals. In fact, they have a low lipid content and a high content of proteins, fibres, unsaturated fatty acids, glucans, vitamins, phenolic compounds, minerals, and secondary metabolites [51,56,57,58]. Numerous studies have shown that edible mushrooms possess anticancer, antiatherosclerotic, hypocholesterolemic, hypolipidemic, antiviral, antimicrobial, immunostimulant, anti-inflammatory, antioxidant, fibrinolytic, and anti-aging properties [11,59,60,61,62,63,64,65,66]. Furthermore, edible and medicinal mushrooms contain several mycochemicals with antioxidant activity, such as phenols, flavonoids, polysaccharides, vitamins, carotenoids, ergothioneine, and others [57,67,68,69] (Figure 2). In recent years, edible mushrooms have attracted more attention for their antioxidant properties, as demonstrated by the large number of papers present in various electronic databases, which is why in the PubMed database, using the keywords “mushrooms and antioxidant” leads to the listing of 3050 resources in the last two decades, 80% of which are from the last ten years.

### 5.1. Polyphenols

Polyphenols are aromatic hydroxylated compounds, structurally characterized by one or more aromatic rings, and ubiquitously present in plants, fruits, and vegetables, which protect against ultraviolet radiation and pathogens. Polyphenols, also, are responsible for the astringency, flavor, and color of foods. Based on the number of phenolic rings, i.e., their structural characteristics, polyphenols can be classified into flavonoids, phenolic acids, stilbenes, and lignans. Flavonoids represent the most studied group of polyphenols, with around 6000 flavonoids [70], and they include six categories of compounds: flavonols, flavones, flavanones, anthocyanins, isoflavones, and flavanols. Structurally, they are diphenylpropanoids (C6-C3-C6), composed of two benzene rings connected by a propanoid chain that forms a heterocyclic ring containing oxygen [51]. The antioxidant activity of polyphenols occurs in several ways. First of all, they can donate electrons, thereby neutralizing ROS and protecting cells against damage. Phenolic compounds are also able to chelate pro-oxidant metals and inhibit enzymes involved in free radical generation [59,70,71,72]. Different studies have demonstrated the ability of flavonoids to protect the brain from neural damage and degeneration in Alzheimer’s disease and dementia [73,74,75]. Several manuscripts report on the presence of polyphenols in mushrooms. Their high variability in the qualitative and quantitative composition shown in various studies could be ascribed to different cultivation methods, environmental conditions, stages of maturation, and genetic differences among species [57]. Phenolic acids, the main phenolic compounds of mushrooms [59,76], are divided into hydroxybenzoic acids (e.g., gallic, p-hydroxybenzoic, syringic, and vanillin) and hydoxycinnamic acids (e.g., p-coumaric, o-coumaric, caffeic, ferulic, and sinapic) [59]. In a recent study, Çayan et al. [77] reported on the identification of 16 phenolic acid compounds from 26 mushrooms collected from Anatolia, showing that fumaric acid was the most abundant phenolic. The authors stated that the total amount of phenolic compounds found was comparable to other foods. Among the most commercially important species, *A. bisporus* (button) and *P. ostreatus* (oyster mushroom) contained different phenolic acids, such as gallic, p-coumaric, cinnamic, caffeic, chlorogenic, ferulic, p-hydroxybenzoic, homogentisic, protocatechuic, catechin, and vanillic [78,79,80]. High concentrations of caffeic acid (approximately 15 μg/g d.w.) can be found in *C. cibarius*, *A. bisporus*, *B. edulis*, *C. gambosa, H. marzuolus*, and *L. deliciosus* [76,78]. As reported by Ferreira et al. [59], different edible mushrooms showed the presence of several flavonoids (e.g., catechin, naringenin, myricetin, and quercetin). Butkhup et al. [81] found (+)-catechin and (−)-epicatechin in 25 mushrooms analyzed. Other phenolic compounds with antioxidant activity, such as quercetin, quercetin-3-O-rutinoside, myricetin, naringenin and kaempferol, were also detected.

### 5.2. Polysaccharides

**Figure 3:**
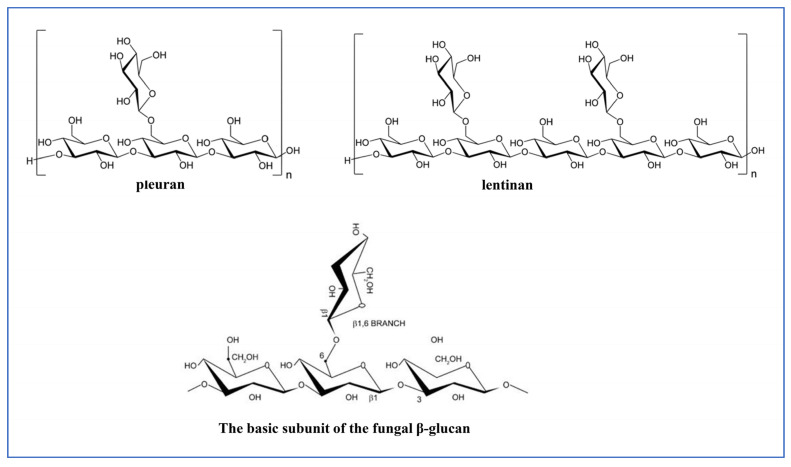
Chemical structure of some fungal polysaccharides.

Polysaccharides, mainly glucans or heteropolysaccharides, deriving from the Basidiomycetes family and medicinal mushrooms, have been known and widely used for their medicinal and functional properties [10,60]. Among fungal polysaccharides, β-glucans are one of the major bioactive constituents and represent a key component of the fungal cell wall. β-glucans are homopolysaccharides formed by β-d-glucose chains linked by glycosidic β-1,3 bond type with β-1,6 glycosidic branches, but some are true heteroglycans containing galactose, glucuronic acid, mannose, xylose arabinose, or ribose. The biological activity of these biopolymers varies as a function of their molecular weight, conformation, solubility in water, type of bond, structure, and characteristics of the side chains, including frequency, position, and length [61] (Figure 3). Most of these are considered fibers since they are not digested by human enzymes. The most well-studied polysaccharides from edible mushrooms are the glucans lentinan, synthetized by *Lentinus edodes* (Shiitake mushroom), schizophyllan that is produced by *Schizophyllum commune*, ganoderan that is produced by *Ganoderma lucidum*, grifolan that is found in *Grifola frondosa*, and pleuran that is found in the oyster mushroom, *Pleurotus ostreatus* [10,82].

Fungal polysaccharides showed biological activity that includes anticancer, anti-inflammatory, hypolipidemic, immunomodulatory, hypoglycemic, antioxidative, and anti-aging [10,60,61,82,83]. In recent years, the antioxidant properties of mushroom polysaccharides have garnered considerable attention, since they are considered to be effective free radical scavengers, reducing agents, and Fe^++^ chelators, also capable of preventing lipid peroxidation and modulating, positively, the activity of antioxidant enzymes such as CAT, SOD, and GPx, which protect living organisms against oxidative damage [10,57,84,85]. Yan et al. [86] compared the antioxidant activities of purified water-soluble polysaccharides from *Pleurotus eryngii*, *Pleurotus ostreatus*, *Flammulina velutipe*, and white *Hypsizygus marmoreus* with the sugar composition and the molecular weight, elucidating the relationship between the structure of mushroom polysaccharide and the antioxidant activity. Various polysaccharides isolated from different mushroom species (*P. eryngii*, *C. comatus*, *G. Lucidum*, *A. mellea*, *G. frondosa*, *R. albonigra*, *G. tsugae*, *F. velutipes*, and *P. ostreatus*) have been shown to exhibit potent antioxidant effects on oxygen radicals [87,88,89,90,91]. In a study by Zou et al. [92], antioxidant polysaccharides obtained from the fruiting bodies of *Auricularia auricula* have shown potent scavenging activity against hydroxyl radicals. The same authors also demonstrated the ability to chelate Fe^++^ by polysaccharides obtained from the fruiting bodies of the mushrooms, *Ganoderma lucidum*, *Agaricus brasiliensis*, *Auricularia auricula*, *Phellinus linteus*, and *Agaricus bisporus* [92]. Polysaccharides extracted from *T. versicolor*, *L. edodes*, *and Agaricus* spp. showed significant chelating properties. The authors correlated this effect with the presence of phenolic molecules, such as ferulic acid and tyrosine, covalently bound to the main chain of glucans [10,11]. Other authors found scavenging activities towards DPPH, superoxide, and hydroxyl radicals [87,88] in polysaccharide fractions extracted from various mushrooms. However, despite the large number of papers demonstrating the antioxidant capacity of mushroom polysaccharides, the mechanism by which they exert this activity is still not fully understood [93]. According to Kishk and Al-Sayed [94], polysaccharides exert their radical scavenging activity with a mechanism similar to that of aromatic compounds, based on the transfer of hydrogen atoms in the case of neutral polysaccharides and of electrons for acid polysaccharides. Among the proposed mechanisms, the introduction of chemical modifications such as phosphorylation, acetylation, sulfation, benzoylation, and carboxymethylation increases the hydrogen donation capacity of polysaccharides by weakening the dissociation energy of hydrogen bond [95,96]. Lo et al. [97] demonstrated that the antioxidant capacity of polysaccharides is modulated by the composition and ratio of monosaccharides as well as type of glycosyl linkage. In addition, the presence of other components such as pigments, phenols, flavones, peptides and proteins bound to polysaccharides might contribute to the increase in antioxidant activity [93].

### 5.3. Antioxidant Vitamins

**Figure 4:**
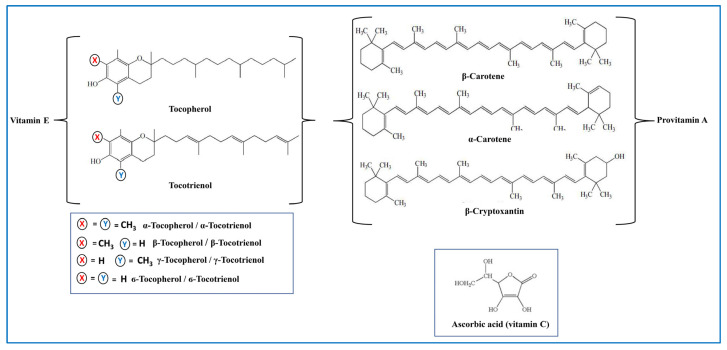
Chemical structure of antioxidant vitamins.

Vitamins are compounds that are essential to the human body for overall normal cell function, growth, and development. Among them, vitamin A, including carotenoids, and vitamins C and E constitute the group of antioxidant vitamins (Figure 4).

Carotenoids are natural lipophilic pigments synthesized from plants, fungi, algae, and several bacteria. Currently, there are some hundred known carotenoids classified as carotenes and xanthophylls, based on their polarity [98]. In diet, as well as in the human body, the most abundant carotenoid compounds are α- and β-carotene, lycopene, β-cryptoxanthin, and lutein [99]. Among them, only β-, α-carotene and β-cryptoxanthin act as provitamin A and can be converted into retinal [100]. Due to the presence of several conjugated double bonds in their chemical structure, carotenoids are efficient antioxidants and radical scavengers [101]. Several studies report the capability of carotenoids to prevent various degenerative disorders related to oxidative stress, such as Alzheimer’s disease and dementia [102,103]. Among the carotenoids, lutein is one of the most studied molecules due to its oxidant scavenging ability [104,105,106]. In elderly individuals, a positive correlation between plasma lutein levels and the reduced risk of Alzheimer’s disease and dementia has been demonstrated [107]. β-carotene, lutein, and lycopene were found in several wild and commercial mushroom edible species such as *Boletus edulis* and *Xerocomus badius* [108]. Zhao et al. [109] reported that environmental stress induced the accumulation of high amounts of antioxidant carotenoids in the edible mushroom *Cordyceps militaris*. In a recent study [110], it was demonstrated, by evaluating the effects of different substrate compositions, that the fruiting body of a cultivated strain of *Pleurotus ostreatus* is capable of accumulating high amounts of vitamin A.

Vitamin C, also known as l-ascorbic acid, is a hydrophilic compound representing the primary antioxidant in plasma and cells, where it is involved in the regulation of redox status in the body. Among all vitamins in the human body, it presents at the highest amount. As an antioxidant, it acts as a reducing agent and an effective radical scavenger, also preventing lipid peroxidation in biomembranes by neutralizing peroxyl radicals [111,112,113,114]. Several studies have demonstrated a correlation between the reduction of tissue levels of vitamin C and the aging process [70]. Other studies have demonstrated the potential protective function of ascorbate in ND, suggesting that low vitamin C levels can influence the redox balance, accelerating the onset of these diseases [115]. Vitamin C was found in several mushroom species [59], showing a high variability in its content due to the effects of the maturating stage of the fruiting body, genotype, weather conditions, and geographic locations [59]. Mishra et al. [116], investigating the antioxidant properties of different edible mushroom species, showed, for the mycelial extracts, an amount of ascorbic acid that was higher in the different *Plerotus* species analyzed. Wild edible mushrooms *Boletus edulis* and *Xerocomus badius* were found to contain means of 22.1 mg and 27.4 mg per 100 g dry weight of ascorbic acid, respectively [108].

Vitamin E is a common term used for a family of eight different chromanol compounds, four α-, β-, γ- and δ- tocopherols, and four corresponding tocotrienols. α- and -γ tocopherols are the two major forms of the vitamin, with the relative proportions of these depending on the source. Among all isoforms, the most biologically active form of vitamin E is α-tocopherol, which is also the predominant form in mammalian tissue. Vitamin E is the major fat-soluble component in the cellular antioxidant defense system. It is localized in cell membranes, where it protects plasma lipoproteins and membrane phospholipids from oxidative damage by scavenging peroxyl radical [117]. Several studies have shown the protective role of vitamin E in neuroinflammation [118,119]. Tocopherols have also been detected in most other mushrooms. Different studies have demonstrated that the amount of vitamin E varied in respect to the mushroom species and the collection site [108,110,120,121]. However, it should be noted that edible mushrooms contain amounts of tocopherols much lower compared to foods rich in vitamin E [11].

### 5.4. Ergothioneine

**Figure 5:**
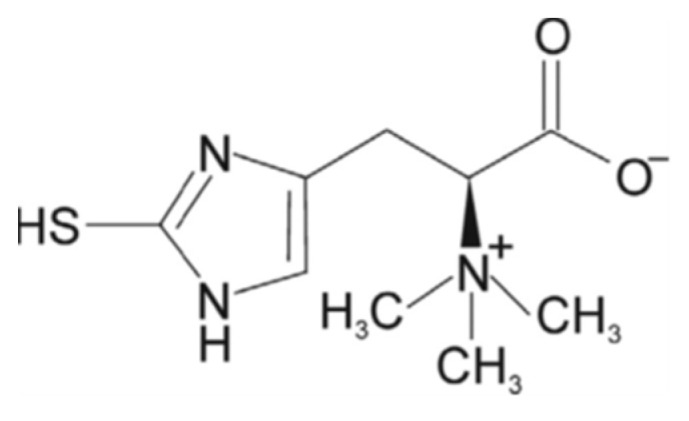
Chemical structure of ergothioneine.

Ergothioneine (ERG) is a thio-amino acid derived from histidine, characterized by a high antioxidant capacity produced from microorganisms, especially from edible mushrooms but also from actinobacteria [122] (Figure 5). Apparoo et al. [123] reported on the role of ergothioneine in promoting longevity through its antioxidant activity, highlighting its capability to modulate both the aging-related signal transduction cascades and gene expression. Several in vivo studies revealed that ERG acts as a potent neuroprotectant in mice exposed to different inducers of neurotoxicity (e.g., cisplatin, beta-amyloid, and d-galactose) by preventing brain lipid peroxidation, increasing GSH levels, and restoring AchE activity, thereby improving learning and memory deficits [124,125,126]. Cheah et al. [127], in a study of a cohort of Asians over 60 years old, observed a significant decrease in the blood levels of ergothioneine. Additionally, in a subset of MCI subjects, plasma ERG levels were significantly lower than those measured in age-matched healthy subjects, suggesting that low levels of ERG may be a risk factor for age-related diseases, such as neurodegenerative disorders. An inverse correlation between age and serum ERG levels were also reported by an epidemiological study conducted in Australia [128]. In another study, ERG has been demonstrated to be effective on cognitive function [129]. ERG levels appear to decrease during neurodegenerative and cardiovascular diseases, which are linked to oxidative stress [130]. ERG has been proposed as a nutritional biomarker for mushroom consumption [131]. As reported by Liu et al. [132], ergothioneine has excellent free radical scavenging ability towards hydrogen peroxide, hydroxyl radicals, and superoxide anions, and is also able to chelate divalent metal ions. Liu et al. [132] demonstrated that approximately 25% of the total antioxidant capacity present in culinary medicinal mushroom extracts was attributable to ERG. The ergothioneine concentration in different species of mushrooms has been found to be significantly different and to vary with physiological or environmental conditions [133,134,135]. Among the most popular mushrooms, those with the highest amounts of ERG include *Pleurotus* spp., [130,136,137], *Lentinula edodes*, *Boletus edulis* [134], and *Agaricus bisporus* [133]. A study carried out on locomotor frailty and cerebellum of aged mice orally supplemented for 2 months with a standardized extract obtained from the mushroom *Hericium erinaceus* demonstrated the neuroprotective effect of *H. erinaceus* metabolites in the prevention and treatment of age-related neurodegenerative diseases [138].

### 5.5. Other Antioxidants

#### 5.5.1. Minerals

Minerals are essential components to maintain physical health. They are also components of enzymes and hormones. All living organisms obtain the necessary intake of minerals for the body through a balanced diet. Basidiomycetes are mushrooms known to be excellent accumulators of minerals in their fruiting bodies from the environment in which they grow. The most abundant mineral elements in mushrooms were K, P, and Mg followed by Na, Ca, Zn, Mn, Fe, Cu, Co, and Ni, whereas selenium is present in all mushrooms, although only a few species can be considered abundant in this element [139,140]. Among minerals, zinc, copper, and selenium possess antioxidant properties.

As an antioxidant, zinc is involved in the regulation of glutathione metabolism, the inhibition of NADPH-oxidase enzyme, and the modulation of metallothionein expression, and it serves as a cofactor for superoxide dismutase enzyme [141,142]. Mushrooms may accumulate high amounts of Zn in their fruiting body [143,144,145]. As well as for other minerals, wild mushrooms are able to accumulate greater quantities of zinc than cultivated species [146,147]. Zheng et al. [148] reported on the edible mushroom *Pholiota nameko* that was used as a vector for the organification of zinc to obtain zinc polysaccharide able to improve in vivo the antioxidant status, indicating a strong anti-aging capability.

Selenium is an essential microelement that displays important antioxidant effects in living organisms. Many of the physiological roles of selenium are due to its presence within selenoproteins, such as GSH-Px, TrxR, and iodothyronine deiodinases [149]. All these enzymes are essential for life and are involved in the control of oxidative stress [150]. Several selenoproteins are antioxidant enzymes displaying anti-aging effects and preventing age-related diseases. Selenium mitigated ROS-mediated inflammation, reduced DNA damage, and prolonged telomere length, thus playing a role in fighting aging and preventing aging-related diseases in the elderly [151]. Mushrooms may accumulate considerable amounts of Se as Se-methyl-l-selenocysteine, a form that is highly bioavailable and directly enters the selenium metabolic pool, thus representing a good way to improve selenium intake [152]. Some authors have demonstrated that mushrooms such as *Pleurotus* spp., *Flammulina velutipes*, *Boletus edulis*, *Agaricus* spp., and *Lycoperdon perlatum* accumulate Se if grown in substrates with a high Se content, while *P. florida*, *Volvariella volvacea*, and *Lentinula edodes* have been found to increase their phenolic profiles and antioxidant status in response to selenium [153,154]. Wild-grown species of edible mushrooms can accumulate Se in a greater amount than cultivated edible mushroom––for example, wild-grown mushrooms of the genus *Agaricus* showed selenium content much greater than the cultivated mushroom [139].

Copper plays a crucial role in human health, and together with Zn is a component of SOD. Copper ions show a high affinity for the human tripeptide GHK, which regulates a large number of human genes involved in neuronal development and maintenance, which suggests the possibility of using GHK as a therapeutic target against cognitive decline and age-dependent neurodegeneration [155]. The ability of GHK to reverse, albeit partially, cognitive impairment through anti-inflammatory and epigenetic pathways has also been demonstrated in an aging mouse model [156]. Mushrooms may accumulate high levels of Zn in their fruiting body [144,156,157,158,159,160].

#### 5.5.2. Glutathione (GSH)

**Figure 6:**
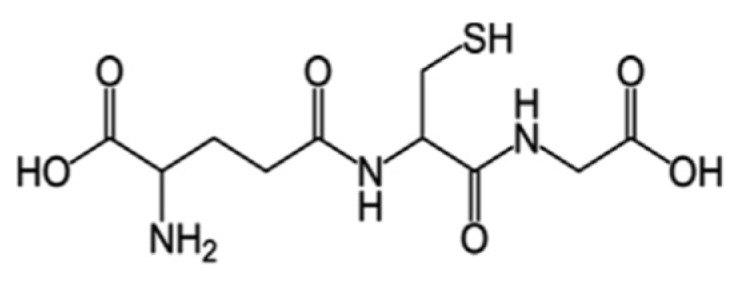
Chemical structure of glutathione.

Glutathione (Figure 6) is a key antioxidant present in microorganisms, plants, and animals, which regulates the redox state of the cell, preventing the damage induced by free radicals, heavy metals, and peroxides. It acts enzymatically through glutathione peroxidase, glutathione reductase, and glutathione-S transferase, and it can also exert its antioxidant activity via non-enzymatic means through the free thiol group of cysteine [161]. The modulation of glutathione metabolism is a useful adjuvant therapy for brain disorders [162]. In particular, aging and various neurodegenerative diseases have been associated with a decrease in GSH levels. It has been demonstrated that alterations of GSH metabolism seem to play a key role in the onset of Parkinson’s disease [163]. It was found that several edible mushroom species, such as *Agaricus bisporus*, *Grifola frondosa*, *Pleurotus ostreatus*, *Lentinula edodes*, *Pleurotus citrinopileatus*, *Agrocybe aegerita*, *Hericium erinaceus*, *Ganoderma lucidum*, *Cantharellus cibarius*, and *Morchella esculenta*, are good sources of glutathione [134,164].

## 6. Antioxidant Compounds of Mushrooms as Neuroprotective Agents

**Figure 7:**
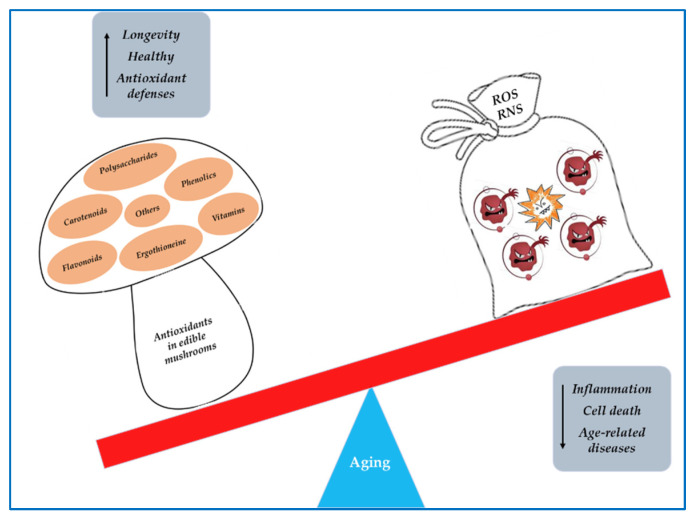
The supplementation of mushroom antioxidants to counteract the ROS/RNS in aging.

It has been reported that mushroom antioxidant compounds, due to their ability of increasing antioxidant defenses and counteract oxidative stress, may prevent age-related neurological diseases [165,166,167,168] (Figure 7).

In this regard, it is worth highlighting that most of the studies conducted to date on in vitro and in animal models that demonstrate the neuroprotective properties of mushrooms have been conducted on mushroom polysaccharides [169] (Table 1).

Cheng et al. [192] showed that polysaccharides purified by the mushroom *Hericium erinaceus* exerted protective effects on Aβ-induced neurotoxicity in PC12 cells through radical scavenging activity, and also reduced ROS production and recovery of mitochondrial function. In another study conducted on glutamatedamaged PC12 cells, Zhang et al. [193] demonstrated that the neuroprotective effect of *H. erinaceus* was associated with the protection of mitochondrial dysfunction and the subsequent suppression of ROS accumulation. Furthermore, the same authors using a mouse model of AD observed an increase in the concentrations of acetylcholine (Ach) and choline acetyltransferase (ChAT) in the serum and hypothalamus of mice treated with *H. erinaceus*. Recently, it has been demonstrated that extracts from *H. erinaceus* possess anti-inflammatory and antioxidant action.

Indeed, the extracts are able to counteract neurotoxicity induced by hydrogen peroxide in HT22 mouse hippocampal neurons by decreasing ROS production and increasing the activity of the antioxidant enzymes CAT and GPx, and by reducing NO levels in LPS-treated BV2 microglia [194]. An et al. [195] demonstrated in an AD mouse model that the administration of polysaccharides derived from *Armillaria mellea* enhanced serum and hypothalamic levels of SOD and GPx and counteracted ROS production. These events were accompanied by an improvement of cholinergic system functions as indicated by the enhancement of Ach and choline ChAT concentration, and by a decrease in acetylcholine esterase (AchE) levels in the serum and hypothalamus. In addition, it was demonstrated that *Armillaria mellea* polysaccharides reduced the deposition of Aβ and attenuated the oxidative damage and p-Tau aggregation in the hippocampus. In a similar study, Zhang et al. [188] showed that the extract of *Pleurotus ostreatus* attenuated learning and memory impairment in an AD rat model elevating SOD, GPx, and CAT activities, and reducing MDA levels and AchE activity. In addition, this extract decreased amyloid peptide formation and Tau phosphorylation by increasing the expression of protein phosphatase 2A and reducing the expression of amyloid precursor protein (APP). The beneficial neurological effects exerted by polysaccharides are not only expressed in pathological conditions but also in the physiological events that characterize aging. In this respect, Chen et al. [181], using a natural aging rat model, demonstrated that polysaccharides from *Grifola frondosa* improved learning function and memory. These improvements were related to the antioxidant properties of *G. frondosa*, as evidenced by the increase in the activity of antioxidant enzymes and total antioxidant capacity, and by the decrease in NO and MDA levels in old rat brain. The neuroprotective and anti-age potential of polysaccharides from *G. frondosa* has been also tested in the yeast cells and *Drosophila melanogaster* models of PD, showing the ability to reduce both ROS levels and the toxicity of synuclein [196].

Although several studies have confirmed the neuroprotective properties of some antioxidant compounds of mushrooms (e.g., polysaccharides) in vitro or in animal models, it will be necessary to verify their effectiveness in various clinical studies in order to support their use in therapies for patients with ND.

## 7. Conclusions and Future Perspectives

Aging is a complex biological process, characterized by an irreversible physical decay that predisposes people to various age-related diseases, including neurodegenerative disorders (ND). Oxidative stress plays a crucial role in the development of age-related ND. It is now widely established by many in vitro and in vivo studies that a protective role is played by antioxidants in the prevention of diseases in which oxidative stress plays a key role. Edible mushrooms, due to their richness in antioxidant compounds such as flavonoids, phenolics, polysaccharides, vitamins, ergothioneine, and others, undoubtedly represent a valid ally in health-promoting strategies. However, it should be noted that there are still very few studies concerning the application of mushroom antioxidants on humans in the context of clinical trials on neuroprotection [197]. Furthermore, the few studies available in humans have been conducted on mushroom extracts/powders or whole mushrooms ingested with the diet [198,199,200,201] and not on isolated antioxidant compounds. Taking into account this aspect, future directions should be aimed towards conducting clinical studies in humans in order to evaluate the neuroprotective potential not only of whole mushrooms but also of the individual antioxidant compounds.

In this study, we have shown that among the various antioxidant compounds of mushrooms, those that have shown a significant neuroprotective effect in studies in vitro and on animal models are polysaccharides and ergothioneine. However, even if there are no specific studies demonstrating that other mushroom antioxidants are able to counteract the neurodegeneration associated with aging, it cannot be excluded that the antioxidative effects evidenced by other mushroom compounds, especially phenolic compounds and vitamins, may exert a synergistic action in counteracting oxidative stress and help maintain a healthy brain. Therefore, future studies should aim to fill this gap by investigating the neuroprotective properties of the other antioxidant compounds of mushrooms, too. 

Generally, edible mushrooms represent very safe and non-toxic foods, obviously excluding cases of individual intolerance or inappropriate preparations (e.g., mushrooms consumed in raw or partially cooked forms). In these cases, mushrooms may cause mild adverse effects such as gastrointestinal and skin reactions. However, the adverse side effects have mostly been seen in cases of excessive mushroom consumption. Therefore, research must also be conducted to define the optimal amounts of the different antioxidant compounds and to standardize the growing conditions and extraction methods in order to provide the maximum benefits [202]. At the same time, research should be aimed at defining any negative effects deriving from the use of mushrooms, such as possible interactions with other compounds or drugs during specific therapeutic treatments.

Furthermore, in order to consider mushroom antioxidant compounds as potential neuroprotective agents, scientific validation is needed and this can be achieved by understanding their molecular and biochemical mechanisms involved in neuroprotection.

## Figures and Tables

**Table 1:** Antioxidant polysaccharides from edible mushrooms with anti-aging effect.

Mushroom Species	Bioactive Compounds/Extracts	Mechanism of Action	References
*Agaricus bisporus*	Acidic-extractable Polysaccharides (AcAPS)	In vitro: hydroxyl and DPPH scavenging activities. In vivo: hepatic and nephric protection by improving serum enzyme activities in aging mice.	[170]
*Agaricus brasiliensis*	Exopolysaccharides (ExPSs) and endopolysaccharides (EnPSs)	In vitro: hydroxyl and DPPH scavenging activities, reducing power. Improvement of total antioxidant capability, decrease in MDA content.	[171]
*Agrocybe aegerita*	Acidic- and alkalic-extractable polysaccharides (Ac-MPS and Al-MPS)	In vitro: hydroxyl and DPPH scavenging activities. In vivo: increase in SOD, CAT, GPx and total antioxidant capacity; decrease in MDA and lipid peroxidation; reduction of serum levels of triglycerides and total cholesterol in aging mice.	[172]
*Agrocybe cylindracea*	Selenium polysaccharides (SL-02) Exopolysaccharides (EPS)	In vitro: hydroxyl and DPPH scavenging activities, reducing power. In vivo: increase in SOD, GPx, and total antioxidant capacity; decrease in MDA and total cholesterol in aging mice.	[173,174]
*Flammulina velutipes*	Sulfated polysaccharides (SFPS)	In vitro: DPPH, hydroxyl, superoxide and scavenging activities; reducing power; Fe^2+^-chelating capacity In vivo: increase in the antioxidant enzyme activities; decrease in lipid peroxidation, improvement of the inflammatory response in mice.	[175]
*Ganoderma lucidum*	Polysaccharides	Reduction of amyloid toxicity; decrease in neurotoxicity; increase in GSH, GPx activities; decrease in MDA. Protection of dopaminergic neurons from inflammation. Inhibition of microglial activation; decrease oin TNF-α and IL-1β.	[176,177,178,179,180]
*Grifola frondosa*	Polysaccharides (GFP) Intracellular zinc polysaccharides (IZPS)	In vitro: hydroxyl, superoxide and DPPH scavenging activities, reducing power. In vivo: increase in SOD, CAT, GPx activities, and total antioxidant capacity; decrease in MDA and nitric oxide levels and amelioration of age-associated changes of brain histology.	[181]
*Hericium erinaceus*	Sulfated residue polysaccharides (SHRPs)	In vitro: scavenging activities. In vivo: increase in enzyme activities, decrease in MDA. Improvement of serum biochemical indices and of immunological activity.	[182]
*Lentinula edodes*	Mycelia polysaccharides (MPS) and mycelia zinc polysaccharides (MZPS)	In vitro: hydroxyl and DPPH scavenging activities, reducing power. In vivo: increase in SOD, GPx, and total antioxidant capacity; decrease in MDA in aging mice.	[183]
*Lepista sordida*	Intracellular polysaccharides (CLSP)	In vitro: hydroxyl, superoxide, and DPPH scavenging activities. In vivo: inhibition of MDA formation; increase in SOD and GPx in aging mice.	[184]
*Pholiota nameko*	Zinc-enriched polysaccharides (MZPS)	Improvement of antioxidant status (SOD, total antioxidant capability, MDA and lipid peroxide) in aging mice.	[148]
*Pleurotus djamor*	Acetylated mycelia polysaccharides (AMPS)	In vitro: hydroxyl, superoxide, and DPPH scavenging activities; reducing power. In vivo: increase in SOD, CAT, and GPx activities; decrease in lipid peroxidation and MDA. Improvement of serum biochemical indices and immunological activity in the liver, kidney, and brain of aging mice.	[185]
*Pleurotus eryngii*	Polysaccharides (PEP) Enzymatic residue polysaccharide (PERP)	Neuroprotective actions against β-amyloid-induced neurotoxicity in cultured rat PC12 cells and aging rats. In vitro: hydroxyl, superoxide and DPPH scavenging activities; reducing power. In vivo: increase in SOD, CAT, GPx activities and total antioxidant capacity; suppression of lipid peroxidation. Improvement of organ functions and histopathological damage in brain, liver, kidney, and skin.	[186,187]
*Pleurotus ostreatus*	Polysaccharides (POP)	Improvement of cognitive impairment in a rat model of Alzheimer’s disease. Increase in SOD, CAT, and GPx activities; reduction of MDA levels and AchE activity.	[188]
*Pleurotus sajor-caju*	Polysaccharide PSP2-1	Improvement of oxidative stress injury, inhibition of apoptosis in H_2_O_2_-induced neuronal cells. Improvement of cognition in aging mice.	[189]
*Tremella fuciformis*	Polysaccharide (TFPS)	Improvement of H_2_O_2_-induced oxidative stress and inhibition of apoptosis in human skin fibroblasts via upregulation of SIRT1 expression.	[190]
*Tricholoma lobayense*	Polysaccharide TLH-3	Inhibition of MDA and increase in SOD and CAT activities in liver and serum of aged mice.	[191]

DPPH: diphenyl-1-picrylhydrazyl; SOD: superoxide dismutase; CAT: catalase; GPx: glutathione peroxidase; MDA: malondialdehyde; TNF-α: tumor necrosis factor-α; IL-1β: interleukin-1β; SIRT1: sirtuin 1; AchE: acetylcholinesterase.

## Data Availability

Not applicable.
